# Combining Cryo-Thermal Therapy with Anti-IL-6 Treatment Promoted the Maturation of MDSCs to Induce Long-Term Survival in a Mouse Model of Breast Cancer

**DOI:** 10.3390/ijms24087018

**Published:** 2023-04-10

**Authors:** Peishan Du, Jiamin Zheng, Shicheng Wang, Yue Lou, Zelu Zhang, Junjun Wang, Yongxin Zhu, Jiaqi You, Aili Zhang, Ping Liu

**Affiliations:** School of Biomedical Engineering and Med-X Research Institute, Shanghai Jiao Tong University, Shanghai 200030, China; dupeishan@sjtu.edu.cn (P.D.); zhengjiamin@sjtu.edu.cn (J.Z.); shichengwang@sjtu.edu.cn (S.W.); louyue4@163.com (Y.L.); zeluzhang1126@sjtu.edu.cn (Z.Z.); sjtuwangjunjun@sjtu.edu.cn (J.W.); zhuyongxin12@sjtu.edu.cn (Y.Z.); jiaqiyou@sjtu.edu.cn (J.Y.); zhangaili@sjtu.edu.cn (A.Z.)

**Keywords:** cryo-thermal therapy, IL-6, mature MDSCs

## Abstract

Immunosuppression plays a significant role in tumor recurrence and metastasis, ultimately causing poor survival outcomes. Overcoming immunosuppression and stimulating durable antitumor immunity are essential for tumor treatment. In our previous study, a novel cryo-thermal therapy involving liquid nitrogen freezing and radiofrequency heating could reduce the proportion of Myeloid-derived suppressor cells (MDSCs), but the remaining MDSCs produced IL-6 by the NF-κB pathway, resulting in an impaired therapeutic effect. Therefore, here we combined cryo-thermal therapy with anti-IL-6 treatment to target the MDSC-dominant immunosuppressive environment, thereby optimizing the efficacy of cryo-thermal therapy. We found that combinational treatment significantly increased the long-term survival rate of breast cancer-bearing mice. Mechanistic investigation revealed that combination therapy was capable of reducing the proportion of MDSCs in the spleen and blood while promoting their maturation, which resulted in increased Th1-dominant CD4^+^ T-cell differentiation and enhancement of CD8^+^ T-mediated tumor killing. In addition, CD4^+^ Th1 cells promoted mature MDSCs to produce IL-7 through IFN-γ, indirectly contributing to the maintenance of Th1-dominant antitumor immunity in a positive feedback loop. Our work suggests an attractive immunotherapeutic strategy targeting the MDSC-dominant immunosuppressive environment, which would offer exciting opportunities for highly immunosuppressive and unresectable tumors in the clinic.

## 1. Introduction

Tumor recurrence and metastasis have become a thorny problem in clinical cancer treatment [1]. Tumor cells recruit a lot of immune cells, and some of them become regulatory after they reach a tumor mass, resulting in an immunosuppressive environment that facilitates tumor recurrence and metastasis [2].

MDSCs are defined as a heterogeneous population of immature myeloid cells and are also recognized as a major obstacle for tumor immunotherapy. The accumulation of MDSCs is highly correlated with advanced stage and a poor outcome of highly aggressive tumors [3,4,5]. Moreover, the high percentage of peripheral MDSCs could serve as a poor indicator for the response of immunotherapy in clinics [6]. MDSCs facilitate immunosuppression through the secretion of suppressive cytokines such as IL-10 and transforming growth factor-β (TGF-β), as well as IL-6, a potent pro-inflammatory cytokine implicated in cancer development [7,8,9], the expression of immune checkpoint molecules, and the products of indoleamine 2,3-dioxygenase (IDO), arginase-1 (Arg-1) and inducible nitric oxide synthase (INOS) [10]. How to overcome MDSC-mediated immunosuppression has become a stringent problem in tumor therapy.

Due to the highly heterogeneous nature of MDSCs in various tumors and the lack of specific targets for MDSCs, it is a significant challenge to remove MDSCs in vivo [11]. Moreover, MDSCs are composed of various myeloid precursor cells that have the capacity to differentiate into dendritic cells and macrophages once they mature [12,13]. Therefore, there are many strategies to induce the differentiation of MDSCs, such as using β-glucan to reprogram MDSCs towards the Antigen-presenting cell (APC) phenotype [14]. As a result, reprogramming MDSCs, rather than depleting them, is a promising strategy for tumor treatment.

IL-6 is more abundant in recurrent metastatic lesions than in primary metastasis [15]. IL-6 upregulates the expression of C-C Motif Chemokine Receptor (CCR)5 in MDSCs by activating the JAK/STAT3 signaling pathway, thus promoting the recruitment of MDSCs [16]. Furthermore, IL-6 induces inhibitory molecules, including programmed death-ligand 1 (PD-L1), Arg-1, and reactive oxygen species (ROS) in MDSCs [17]. At the same time, IL-6 downregulates the expression of major histocompatibility complex class II (MHC-II) molecules, ultimately leading to an enhancement of the inhibitory function exerted by MDSCs [17]. Presently, IL-6 is regarded as a major regulator of MDSC activity and a possible target for cancer immunotherapy [17]. However, anti-IL-6 alone has no significant efficacy in many aggressive cancers, including multiple myeloma, metastatic renal cell carcinoma, prostate cancer and non-small cell lung cancer [18,19]. Therefore, combining anti-IL-6 treatment with other antitumor therapies could be a promising therapeutic approach.

In our previous study, a novel cryo-thermal therapy could disrupt tumor cells in situ through alternating cooling and heating. Moreover, it stimulated a durable antitumor immune response by releasing large amounts of tumor neoantigens and damage-associated molecular patterns (DAMPs) [20,21]. Cryo-thermal therapy could improve the long-term survival of tumor-bearing mice, but the survival rate of the breast cancer-bearing mice was only approximately 50% [22], which was significantly lower than that of the B16F10 melanoma-bearing mice (approximately 80%) [20]. 4T1 cells share many molecular features with human triple-negative breast cancer [23], which is highly aggressive and metastatic [24]. In particular, mice bearing 4T1 breast cancer exhibit the pronounced expansion and accumulation of MDSCs in peripheral blood, spleen and primary tumors [25,26]. As a result, it is critical to investigate how to improve the survival rate of tumor-bearing mice with MDSC-dominated immunosuppressive environment after cryo-thermal therapy.

In this study, we found that after cryo-thermal therapy, MDSCs produced IL-6 by the NF-κB pathway to maintain their immunosuppressive capabilities, impairing the therapeutic effect of treatment. To address this limitation, cryo-thermal therapy in conjunction with anti-IL-6 treatment was used to further promote the maturation of MDSCs, ultimately improving the long-term survival of mice with breast cancer. Our findings revealed that combination therapy reprogrammed MDSCs into a more mature phenotype, resulting in CD4^+^ Th1-dominant differentiation and enhancement of the cytotoxic function of CD8^+^ T cells. Moreover, CD4^+^ Th1 cells helped mature MDSCs produce IL-7 via the IFN-γ/IRF-1 axis, which helped to sustain CD4^+^ Th1-dominant differentiation and further improve the long-term antitumor immunity induced by cryo-thermal therapy. Overall, our present studies indicated that combination therapy was a reasonable treatment for MDSC-dominant immunosuppressive tumors and provided a promising and feasible combined therapeutic strategy for unresectable locally advanced or metastatic cancer in clinical trials.

## 2. Results

### 2.1. Cryo-Thermal Therapy Alone Decreased the Proportion of MDSCs in the Spleen and Blood but Induced the Production of IL-6 in MDSCs through the NF-κB Pathway

**Figure 1:**
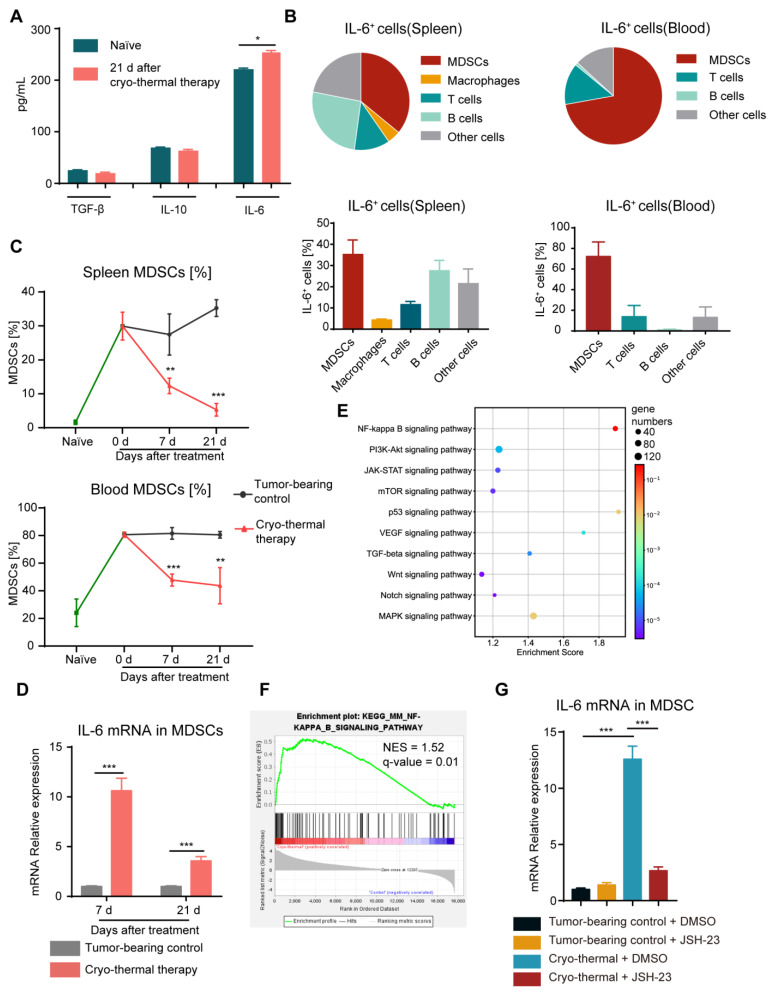
MDSCs activated the NF-κB signaling pathway to stimulate the production of IL-6. (**A**) The serum levels of TGF-β, IL-10 and IL-6 were determined using ELISA on Day 21 after cryo-thermal therapy. (**B**) The major constituent cells of the IL-6^+^ cells in the spleen (left) and blood (right) on Day 21 after cryo-thermal therapy were detected by flow cytometry. (**C**) The proportion of MDSCs (CD11b^+^Gr-1^+^) in the spleen and blood was detected on Day 7 and Day 21 after cryo-thermal therapy. (**D**) The expression of *IL-6* in MDSCs from the spleen at different time points after cryo-thermal therapy was detected by quantitative real-time PCR (qRT-PCR). *n* = 4 for each group. (**E**) Bubble map of KEGG enrichment signaling pathway in splenic MDSCs. (**F**) NF-κB signaling pathway in splenic MDSCs, NES represented normalized enrichment score, *n* = 3 for each group. (**G**) The mRNA level of *IL-6* in MDSCs from the spleen with or without treatment with JSH-23 (10 μM). *n* = 4 for each group. * *p* < 0.05, ** *p* < 0.01, *** *p* < 0.001. Data were analyzed using one-way ANOVA (comparing three or more experimental groups) and two-sided Student’s *t*-test (comparing two experimental groups).

Our previous study demonstrated that cryo-thermal therapy alone failed to raise the survival rate of the mice with 4T1 breast cancer to levels consistent with the mice with B16F10 melanoma, potentially due to the incomplete reversal of the MDSC-mediated immunosuppressive state. Chronic inflammatory cytokines such as IL-6, IL-10 and TGF-β were found to be important in mediating the expansion and suppressive functions of MDSCs, resulting in tumor angiogenesis, development and metastasis [7,8,9,10]. Then, the concentrations of TGF-β, IL-10 and IL-6 in serum were measured on Day 21 after cryo-thermal therapy by ELISA. Compared to the naïve group, there was no obvious difference in the serum levels of TGF-β and IL-10 after cryo-thermal therapy (Figure 1A). However, a high serum concentration of IL-6 was maintained on Day 21 after cryo-thermal therapy. Moreover, the concentration of IL-6 was the highest among all three cytokines (Figure 1A), implying that IL-6 is the major regulator in modulating the therapeutic effect of cryo-thermal therapy. To ascertain the major source of IL-6, IL-6^+^ cells in spleen and blood after treatments were analyzed by using flow cytometry. The results showed that the majority of IL-6^+^ cells were MDSCs, which accounted for approximately 35% of IL-6^+^ cells in the spleen and nearly 70% of IL-6^+^ cells in the blood (Figure 1B).

MDSCs are the most important immunosuppressive cells in the 4T1 murine breast cancer model, contributing to the immunosuppressive environment [27]. Therefore, we quantified the percentage of MDSCs in spleen and blood of 4T1 subcutaneous breast cancer in the different stages after cryo-thermal therapy by using flow cytometry. Compared to naïve mice, significantly higher levels of MDSCs in tumor-bearing mice accumulated in spleen and blood (Figure 1C). Compared to tumor-bearing mice, the proportion of MDSCs was significantly reduced at all time points after cryo-thermal therapy. However, high proportion of MDSCs remained in spleen and blood after treatment compared to naïve mice (Figure 1C). Moreover, the mRNA level of IL-6 in MDSCs from the spleen after cryo-thermal therapy was significantly upregulated at both the early (7 d) and late (21 d) stages compared with that in tumor-bearing mice (Figure 1D). These results suggested that despite the downregulated proportion of MDSCs after cryo-thermal therapy, there was still residual MDSCs accompanied by the increased expression of IL-6 after treatment, which would impair the therapeutic effect of cryo-thermal therapy in the 4T1 breast cancer model.

Then, to investigate the change in MDSCs after cryo-thermal therapy, we analyzed RNA-seq data from previous studies [28]. Activated signaling pathways were studied using the kyoto encyclopedia of genes and genomes (KEGG) and gene-set enrichment analysis (GSEA), which was based on the NF-κB signaling pathway (mmu04064). Among all the signaling pathways, the NF-κB signaling pathway in MDSCs from the spleen was the most enriched (Figure 1E,F). NF-κB directly binds to the promoter region of IL-6 and facilitates its expression, which involves the acquisition of a protumoral immunosuppressor TAM [29,30]. To confirm the role of NF-κB signaling activation in regulating IL-6 production in MDSCs, JSH-23, which is an inhibitor of NF–κB, was used to interfere with NF-κB nuclear translocation. As shown in Figure 1G, via NF-κB pathway blockade, the expression of *IL-6* in splenic MDSCs was significantly reduced after cryo-thermal therapy compared with that in tumor-bearing mice. These results implied that, despite the decreased proportion of MDSCs in the spleen and blood, NF-κB signaling was activated in residual MDSCs, leading to a high level of IL-6 production after cryo-thermal therapy.

### 2.2. Combining Cryo-Thermal Therapy with Anti-IL-6 Treatment Promoted the Maturation of MDSCs

**Figure 2:**
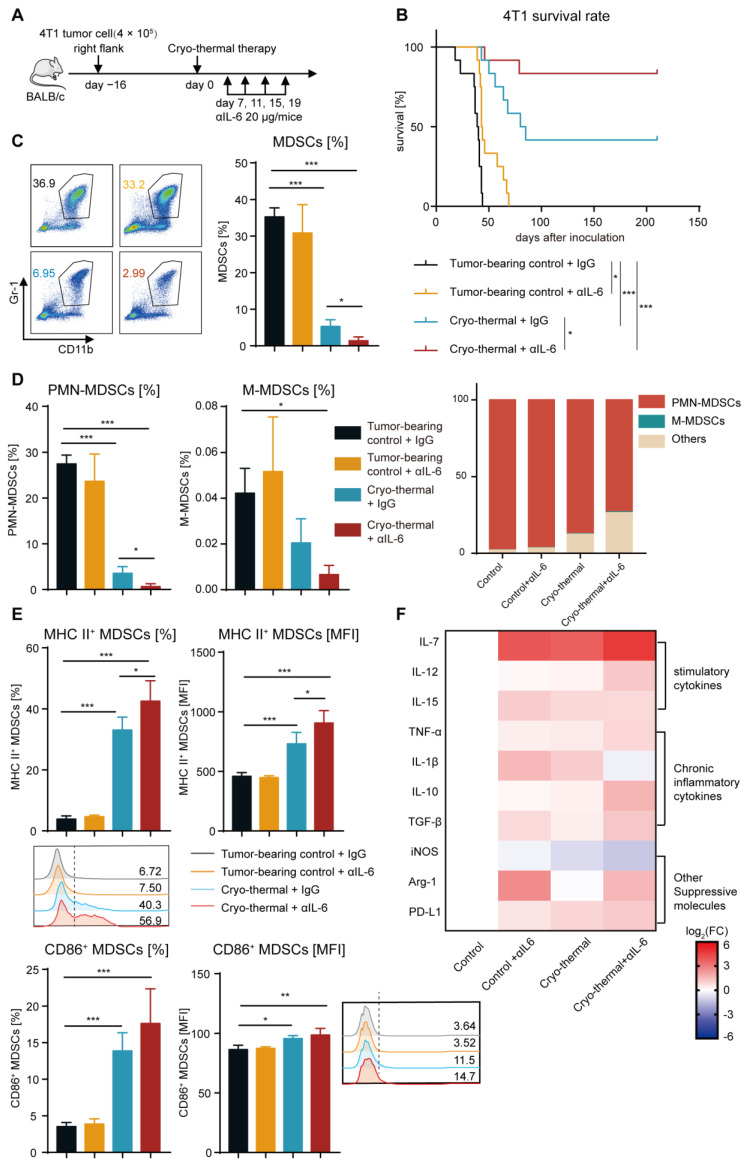
Combination therapy increased the survival rate of 4T1 breast cancer-bearing mice, reduced MDSC accumulation, and promoted MDSC maturation in the spleen. (**A**) Scheme of experiment design. (**B**) A Kaplan–Meier survival curve showed 4T1 breast cancer-bearing mice either treated with single anti-IL-6 mAb, cryo-thermal therapy or combination therapy, *n* = 12 per cohort, log-rank test was used to compare survival curve. (**C**,**D**) The proportion of MDSCs (**C**), PMN-MDSCs and M-MDSCs (**D**) in the spleen from tumor-beaing control + IgG (black), tumor-beaing control + αIL-6 (orange), cryo-thermal + IgG (blue), cryo-thermal + αIL-6 (red). (**E**) The mature phenotype of splenic MDSCs. (**F**) The mRNA levels of stimulatory and inhibitory genes in splenic MDSCs were analyzed by qRT-PCR *n* = 4 for each group. * *p* < 0.05, ** *p* < 0.01, *** *p* < 0.001. Data were analyzed using one-way ANOVA.

To investigate whether the elevated level of IL-6 played an important role in affecting the survival rate of the 4T1 breast cancer-bearing mice after cryo-thermal therapy, mice bearing 4T1 subcutaneous tumor on Day 16 after implantation were treated with cryo-thermal therapy, and 20 μg of anti-IL-6 mAb or isotype IgG mAb was injected i.p. from Day 7 after cryo-thermal therapy (Figure 2A). Anti-IL-6 treatment alone prolonged the survival time but did not improve the survival rate of the 4T1 breast cancer-bearing mice. The survival rate of mice was nearly 41% after cryo-thermal therapy, but combining cryo-thermal therapy with anti-IL-6 treatment (combination therapy) led to a survival rate of approximately 83% (Figure 2B). These data suggested that the elevated level of IL-6 attenuated the survival of the 4T1 breast cancer-bearing mice after cryo-thermal therapy, and combination therapy could significantly improve the survival of the 4T1 breast cancer-bearing mice. These results suggested that combination therapy has the potential to improve the survival rate of 4T1 breast cancer-bearing mice.

On the basis of above results, we hypothesized that the remaining MDSCs promoted their immunosuppressive activity by autocrine IL-6 after cryo-thermal therapy alone. Then, the percentage and phenotype of MDSCs were detected on Day 21 after treatment. The percentage of MDSCs in the spleen and blood was not different after anti-IL-6 treatment alone when compared to the tumor-bearing group (Figure 2C and Figure S3A). However, the proportion of MDSCs in the spleen and blood significantly decreased after cryo-thermal therapy, and this decrease was even more pronounced in the combination therapy group (Figure 2C and Figure S3A). These data suggested that combination therapy and cryo-thermal therapy alone could inhibit the expansion of MDSCs. MDSCs (CD11b^+^Gr1^+^ cells) were further distinguished based on the markers of granulocytic Ly6G and monocytic Ly6C. PMN-MDSCs were the predominant populations in MDSCs among all groups, comprising over 60% of MDSCs in the combination therapy group, and they were even more abundant in other groups, particularly in the tumor-bearing control group (approximately 90%) (Figure 2D). In comparison to the tumor-bearing control group, the percentage of splenic PMN-MDSCs was decreased after cryo-thermal therapy, but anti-IL-6 treatment alone had no effect on them (Figure 2D). After combination therapy, the proportion of PMN-MDSCs was further reduced compared with that after cryo-thermal therapy (Figure 2D). The proportion of splenic M-MDSCs was reduced compared to the tumor-bearing group (Figure 2D). These findings implied that PMN-MDSCs played a major role in the 4T1 subcutaneous tumor model, which could be markedly impaired after combination therapy.

The maturation of MDSCs, which are characterized by the expression of MHC II or CD86, leads to a reduction in their suppressive activity and contributes to the reversal of the suppressive immune environment [31,32,33]. Thus, the frequency and MFI of MHC II and CD86 on MDSCs were measured by flow cytometry after treatment. They were not significantly different after anti-IL-6 treatment alone compared with those in the tumor-bearing control group (Figure 2E and Figure S3B). However, the elevated expression levels of MHC II on MDSCs were induced in the spleen and blood after cryo-thermal therapy, and combination therapy further elevated the frequency of MHC II^+^ MDSCs (Figure 2E and Figure S3B). The proportion of CD86^+^ MDSCs in the spleen and blood after combination therapy was much higher than that in the tumor-bearing control group, and there was an increasing trend compared to the cryo-thermal therapy group (Figure 2E and Figure S3C). On the basis of the above results, we hypothesized that combination therapy would trigger the distinct gene expression profiles of MDSCs. To verify this hypothesis, splenic MDSCs were sorted by using magnetic-activated cell sorting (MACS), and the gene expression profiles of MDSCs (including the stimulatory cytokines *IL-7*, *IL-12* and *IL-15*; the chronic inflammatory cytokines *TNF-α*, *IL-1β*, *IL-10* and *TGF-β*; and other suppressive molecules *iNOS*, *Arg-1* and *PD-L1*) were analyzed by qRT-PCR. Anti-IL-6 treatment alone increased the expression of stimulatory cytokines (*IL-7* and *IL-15*) in MDSCs compared to the tumor-bearing control (Figure 2F). However, *IL-1β*, which is considered a chronic inflammatory cytokine and correlates with MDSC expansion and activity [34], as well as the suppressive molecule Arg-1, were also elevated (Figure 2F). Furthermore, compared to the tumor-bearing control, cryo-thermal therapy upregulated the expression of both stimulatory cytokines and suppressive molecules in MDSCs, except for unchanged levels of Arg-1 and IL-12 and downregulation of iNOS (Figure 2F). Combination therapy led to a further proinflammatory phenotype of MDSCs, reflected in the increased expression of IL-7 and IL-12 in MDSCs, along with the downregulated expression of the suppressive molecule (iNOS) and the chronic inflammatory cytokine (IL-1β) compared with cryo-thermal therapy (Figure 2F). Taken together, these results demonstrated that combination therapy mediated the reprogramming of MDSCs, as evidenced by decreasing the proportion of MDSCs in the spleen and blood and promoting the phenotypic and functional maturation of MDSCs.

### 2.3. Combination Therapy Promoted the Differentiation of MDSCs to Macrophages and the Maturation of Macrophages

**Figure 3:**
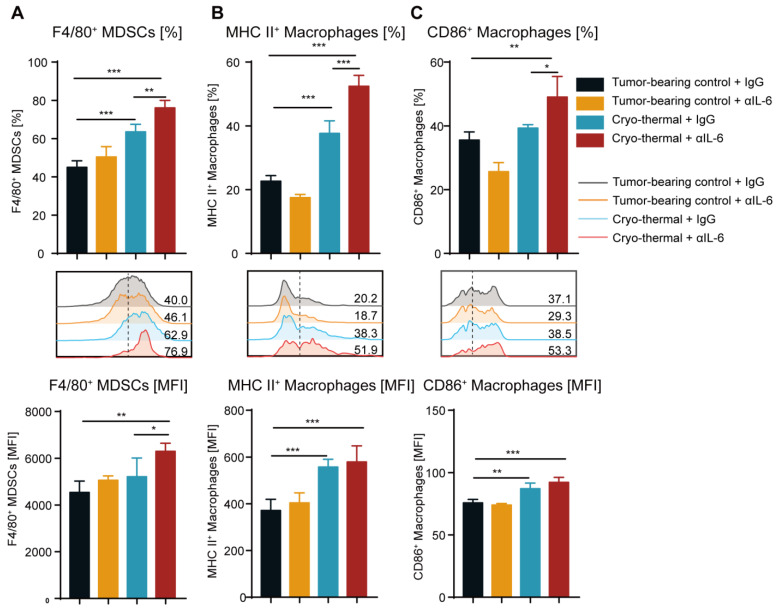
Combination therapy induced the differentiation of splenic MDSCs to macrophages and enhanced their maturation. (**A**) The frequency and MFI of F4/80 on splenic MDSCs. (**B**,**C**) The frequency and MFI of MHC II^+^ (**B**) and CD86^+^ (**C**) macrophages in the spleen were detected by flow cytometry. *n* = 4 for each group. * *p* < 0.05, ** *p* < 0.01, *** *p* < 0.001. Data were analyzed using one-way ANOVA.

Among the hallmark characteristics of mature MDSCs are the increased expression levels of F4/80 (the differentiation marker of macrophages) and CD11c (the differentiation marker of DCs) [13,35]. Therefore, markers of the differentiation of MDSCs into macrophages (F4/80) and DCs (CD11c) were detected using flow cytometry. Anti-IL-6 treatment alone did not change the expression of F4/80 on splenic MDSCs compared to that in the tumor-bearing group, while the higher frequency of splenic F4/80^+^ MDSCs was induced after cryo-thermal therapy (Figure 3A). In addition, the expression of F4/80 on splenic MDSCs was significantly increased after combination therapy compared with that after cryo-thermal therapy alone (Figure 3A). Furthermore, combination therapy increased the frequency of CD11c^+^ MDSCs in the spleen compared to the tumor-bearing control and cryo-thermal therapy (Figure S4A), which suggested that combination therapy markedly promoted MDSCs to differentiate into macrophages and DCs. IL-6 enhances tumor progression by inducing a less mature phenotype of macrophages [36]. Thus, the mature phenotype of macrophages in the spleen was analyzed. After anti-IL-6 treatment alone, there was no difference in the expression of MHC II or CD86 on macrophages compared with that in the tumor-bearing group (Figure 3B,C). In contrast, the high frequency of MHC II^+^ macrophages in the spleen after cryo-thermal therapy was observed compared with that in tumor-bearing mice (Figure 3B). Moreover, the combination therapy markedly induced the frequency of MHC II^+^ or CD86^+^ macrophages in the spleen in comparison with cryo-thermal therapy (Figure 3B,C). The mature phenotype of DCs was not changed after combination therapy (Figure S4B,C). Cumulatively, our findings implied that cryo-thermal therapy promoted the differentiation of MDSCs into macrophages and enhanced the maturation of macrophages, and combination therapy further facilitated this transition.

### 2.4. Combination Therapy Enhanced the Accumulation and Anti-Tumorigenic Capabilities of T Cells

**Figure 4:**
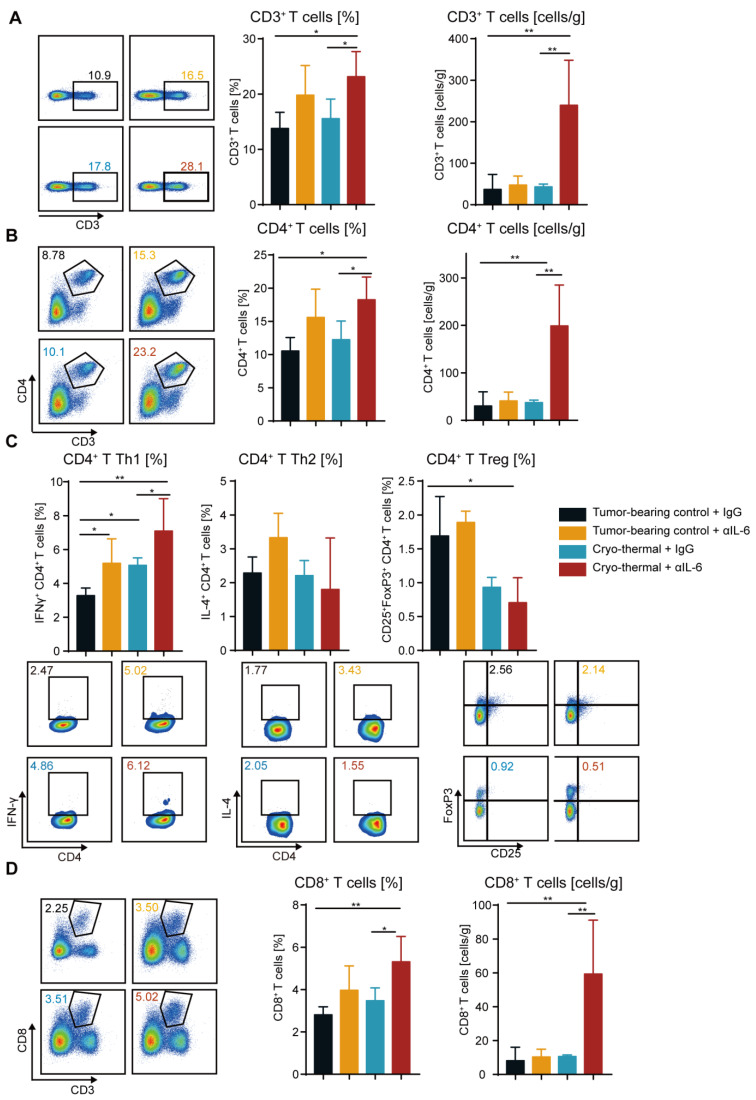
Combination therapy promoted the increase and antitumor activities of T cells. (**A**,**B**) The proportion and absolute number of CD3^+^ T cells (**A**), CD4^+^ T cells (**B**) in the spleen were analyzed by flow cytometry. (**C**) The subsets of CD4^+^ T cells in the spleen. (**D**) The percentage and absolute number of CD8^+^ T cells in the spleen. In the representative graph of flow cytometry, black represent tumor-beaing control + IgG, orange represent tumor-beaing control + αIL-6, blue represent cryo-thermal + IgG, red represent cryo-thermal + αIL-6. *n* = 4 for each group * *p* < 0.05, ** *p* < 0.01. Data were analyzed using one-way ANOVA.

The above studies revealed that combination therapy significantly improved the survival rate of the mice with 4T1 breast cancer, which depended on the decreased proportion and increased maturation of MDSCs in the spleen and blood. MDSCs play an essential role in the inhibition of T-cell activity, and T cells are the key factor mediating antitumor immunotherapy [37]. Hence, it is crucial to investigate the impact of combination therapy on the proportion and function of T cells. The numbers of CD3^+^, CD4^+^ and CD8^+^ T cells in the spleen and blood were assessed by flow cytometry. Compared with those in the tumor-bearing and cryo-thermal therapy groups, the proportion of splenic CD3^+^, CD4^+^ and CD8^+^ T cells after combination therapy was significantly increased. (Figure 4A–D). Moreover, the percentage of CD3^+^, CD4^+^ and CD8^+^ T cells in the blood increased after cryo-thermal therapy compared with those in the tumor-bearing control (Figure S5A–C). Combination therapy maintained a high proportion of T cells after cryo-thermal therapy alone (Figure S5A–C). Furthermore, the absolute numbers of CD3^+^, CD4^+^ and CD8^+^ T cells in the anti-IL-6 treatment and cryo-thermal therapy groups were not significantly different from those in the tumor-bearing group (Figure 4A–D and Figure S5A–C). However, combination therapy significantly increased the absolute numbers of T cells in the spleen and blood (Figure 4A–D and Figure S5A–C). These findings demonstrated that combination therapy significantly promoted the accumulation of T cells. Our previous work demonstrated that cryo-thermal therapy significantly promoted Th1-dominant CD4^+^ T cells to induce durable and robust antitumor immunity [20]. Therefore, splenic CD4^+^ T subsets were detected by using flow cytometry. As shown in Figure 4C, the percentage of CD4^+^ Th1 was increased after anti-IL-6 treatment and cryo-thermal therapy compared with that in tumor-bearing control. Meanwhile, after combination therapy, a higher population of CD4^+^ Th1 subsets was observed compared to the tumor-bearing control and cryo-thermal therapy groups (Figure 4C), which suggested that combination therapy further enhanced Th1-dominant CD4^+^ T-cell differentiation induced by cryo-thermal therapy. After combination therapy, the proportion of Th17 cells was maintained at the same level as that in the tumor-bearing control group, while Th17 cells were slightly increased after cryo-thermal therapy (Figure S6A). In addition, after anti-IL-6 treatment and cryo-thermal therapy, a higher percentage of Tfh was observed compared to the tumor-bearing control group, while combination therapy significantly reduced it to the lowest level among the four groups (Figure S6B). The frequency of Th2 and PD-1^+^ CD4^+^ T cells were not obviously different in each group (Figure 4C and Figure S7A). Notably, the percentage of splenic Tregs CTLA-4^+^ and Tim-3^+^ CD4^+^ T cells were significantly decreased after combination therapy compared with those in the tumor-bearing control group (Figure 4C and Figure S7B,C), indicating that the suppressive CD4^+^ T subsets and exhausted CD4^+^ T cells were significantly reduced after combination therapy. In addition, combination therapy did not alter the percentage of IFN-γ^+^, granzyme B^+^ and perforin^+^ CD8^+^ T cells compared with cryo-thermal therapy (Figure S8B). Combination therapy did not affect the proportion of PD-1^+^ CD8^+^ T cells but reduced the frequency of CTLA-4^+^ and Tim-3^+^ CD8^+^ T cells as compared to the tumor-bearing group (Figure S7D–F). Overall, these data indicated that combination therapy further enhanced Th1-dominant antitumor immunity induced by cryo-thermal therapy and inhibited the differentiation of Tregs, Th17 and Tfh, as well as CD4^+^ T exhaustion, which contributed to a durable antitumor immune response, leading to a significant improvement in the survival rate of the 4T1 breast cancer-bearing mice.

### 2.5. Combination Therapy-Induced CD4^+^ T Cells Enhanced Cytotoxic Function of CD8^+^ T Cells

**Figure 5:**
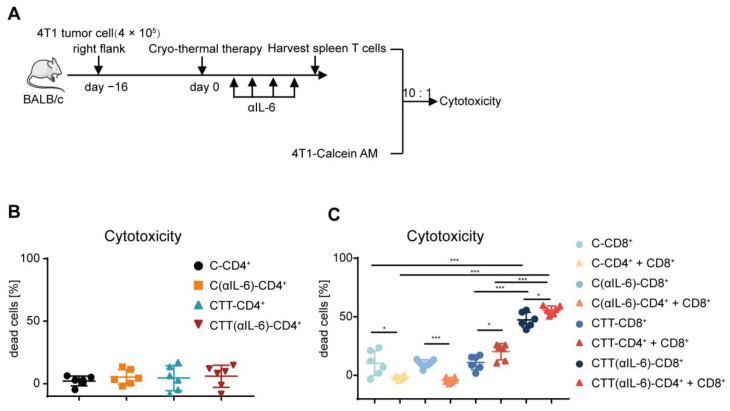
Tumor-killing ability of CD8^+^ T cells was mediated by CD4^+^ T cells. (**A**) 4T1 cells were labeled with 1 μM calcein-AM, and then co-cultured with splenic CD4^+^ and CD8^+^ T cells isolated by MACS at the E: T ratio of 10:1, six hours later, the cytotoxicy of T cells was analyzed by microplate reader. (**B**,**C**) The cytotoxicy of CD4^+^ T cells (**B**), and CD4^+^ T cells combined with CD8^+^ T cells (**C**). *n* = 6 for each group. * *p* < 0.05, *** *p* < 0.001. Data were analyzed using one-way ANOVA. C, tumor-bearing control; CTT, cryo-thermal therapy.

To further identify the principal function of the increased T cells after combination therapy, the killing ability of cytotoxic T cells on target tumor cells was studied. Splenic CD4^+^ and CD8^+^ T cells were isolated using MACS and cocultured with 4T1 murine mammary cancer cells labeled with calcein-AM. Six hours later, the fluorescence level of calcein in the supernatant was measured as a marker for the cytotoxicity of T cells. Then, the killing abilities of CD4^+^ and CD8^+^ T cells on target 4T1 tumor cells were studied (Figure 5A). CD4^+^ The T-mediated killing of tumor cells was not observed in all groups (Figure 5B), indicating that CD4^+^ T cells did not directly participate in killing tumor cells. However, CD8^+^ T cells after cryo-thermal therapy displayed higher cytotoxicity to 4T1 cells than those from tumor-bearing control (median cytotoxicity 12.02% versus 9.59%) (Figure 5C). After combination therapy, the cytotoxicity of CD8^+^ T cells was significantly enhanced compared to that in the cryo-thermal therapy group (median cytotoxicity 46.27% versus 12.02%) (Figure 5C). The above results indicated that CD8^+^ T cells after combination therapy displayed a strong capacity to kill tumor cells, CD4^+^ T cells had no such effect. Then, we hypothesized that CD4^+^ T cells modulate the cytotoxic capability of CD8^+^ T cells. Thus, CD4^+^ T cells from the four groups were isolated by MACS, and the cytotoxic capability of the corresponding CD8^+^ T cells was further analyzed after coculturing with CD4^+^ T cells. As shown in Figure 5C, the addition of CD4^+^ T cells from the cryo-thermal therapy group significantly increased the susceptibility of 4T1 cells to the cytotoxicity of the corresponding CD8^+^ T cells (Figure 5C). Importantly, in the combination therapy group, the addition of CD4^+^ T cells resulted in a greater cytotoxic capacity of CD8^+^ T cells (Figure 5C). However, CD4^+^ T cells from the tumor-bearing and anti-IL-6 treatment alone groups reduced the cytotoxicity of the corresponding CD8^+^ T cells (Figure 5C), which further demonstrated that CD4^+^ T cells from combination therapy could enhance the cytotoxic function of CD8^+^ T cells.

### 2.6. Reprogramming MDSCs toward a Mature Phenotype after Combination Therapy Restored the Antitumor Activity of Other Immune Cells

**Figure 6:**
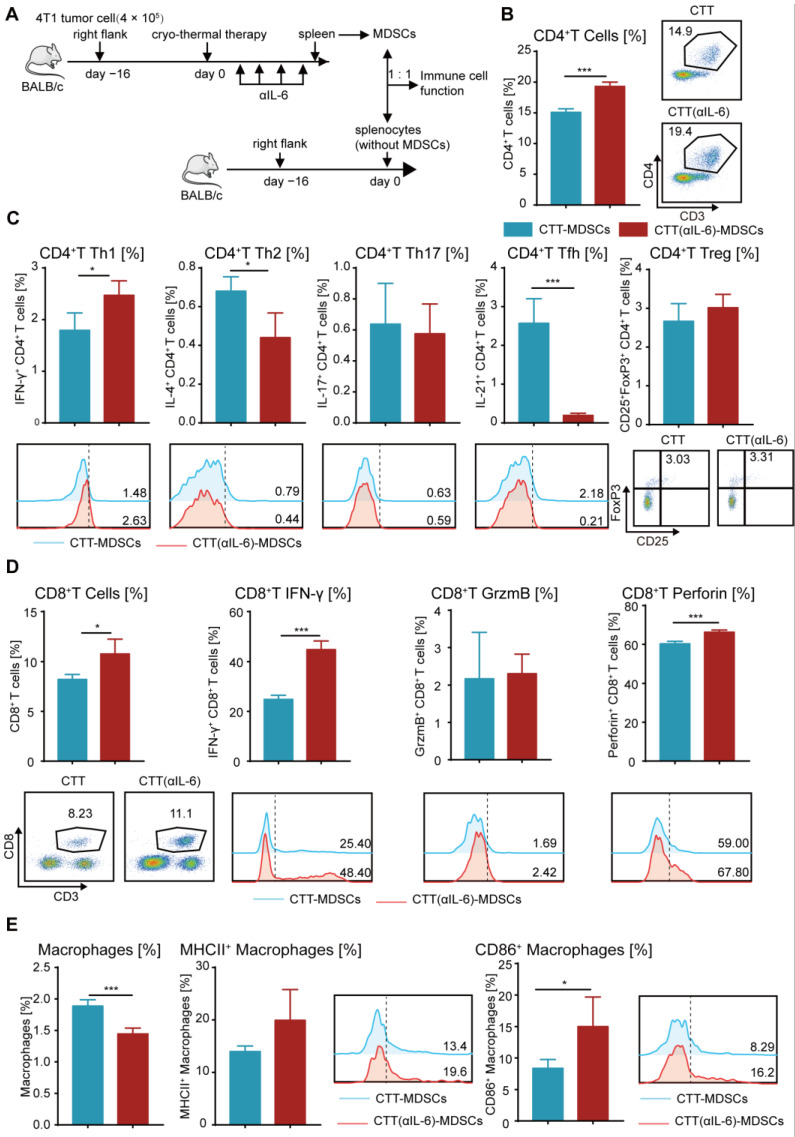
Mature MDSCs after combination therapy promoted the antitumor function of other immune cells. (**A**) Scheme of experiment design. (**B**) The proportion of CD4^+^ T cells. (**C**) The subsets of CD4^+^ T cells. (**D**) The proportion and cytokines of CD8^+^ CTLs. (**E**) The proportion and mature phenotype of macrophages after coculture with MDSCs from different groups in vitro. *n* = 4 for each group. * *p* < 0.05, *** *p* < 0.001. Data for graphs were calculated using two-sided Student’s *t*-test. CTT, cryo-thermal therapy.

The above results indicated that combination therapy further promoted the differentiation of CD4^+^ Th1 subsets and enhanced the tumor-killing capacity of T cells. Given that combination therapy could reduce MDSC accumulation and enhance the maturation of MDSCs, we postulated that the strong antitumor function of T cells would be triggered by mature MDSCs after combination therapy. Thus, splenic MDSCs from the cryo-thermal and combination therapy groups were isolated by MACS and cocultured in a 1:1 ratio with splenocytes from tumor-bearing control mice (without MDSCs) for 24 h, and the percentage and phenotype of T cells were detected by flow cytometry (Figure 6A). As illustrated in Figure 6B,C, the percentage of total CD4^+^ T cells (Figure 6B) and the Th1 subset (Figure 6C) was significantly increased, while Th2 and Tfh cells were significantly decreased when cocultured with MDSCs after combination therapy compared to those from the cryo-thermal therapy group (Figure 6C). Meanwhile, when cocultured with MDSCs from the combination therapy group, the percentage of Th17 cells and Tregs was the same as those in the coculture with those from the cryo-thermal therapy group (Figure 6C). These results indicated that the mature MDSCs from combination therapy could promote the differentiation of CD4^+^ Th1 cells and inhibit CD4^+^ T-cell differentiation into Th2 and Tfh cells. Moreover, the frequency of IFN-γ^+^ and perforin^+^ CD8^+^ T cells were significantly increased when cocultured with MDSCs from combination therapy compared with those from cryo-thermal therapy (Figure 6D). The frequency of granzyme B^+^ CD8^+^ T cells was not changed when the cells were cocultured with MDSCs from the different groups (Figure 6D). These results suggested that the mature MDSCs from combination therapy promoted the cytotoxic function of CD8^+^ T cells.

MDSCs can modify the phenotype and antigen-presenting capability of macrophages [38]. Compared with MDSCs from the cryo-thermal therapy group, MDSCs from the combination therapy group induced a significant increase in CD86^+^ macrophage and a slight increase in MHC II^+^ macrophage (Figure 6E), suggesting that the mature MDSCs after combination therapy stimulated the maturation of macrophages, thereby reshaping the immune environment of mice.

Taken together, these in vitro studies suggested that combination therapy reprogrammed MDSCs to a mature phenotype, which promoted the maturation of macrophages and enhanced the antitumor capacity of T cells, contributing to improving the survival of the mice with 4T1 breast cancer after combination therapy.

### 2.7. Mature MDSCs after Combination Therapy Promoted CD4^+^ T-Cell Proliferation Partly through the IFN-γ/IRF-1/IL-7 Axis

**Figure 7:**
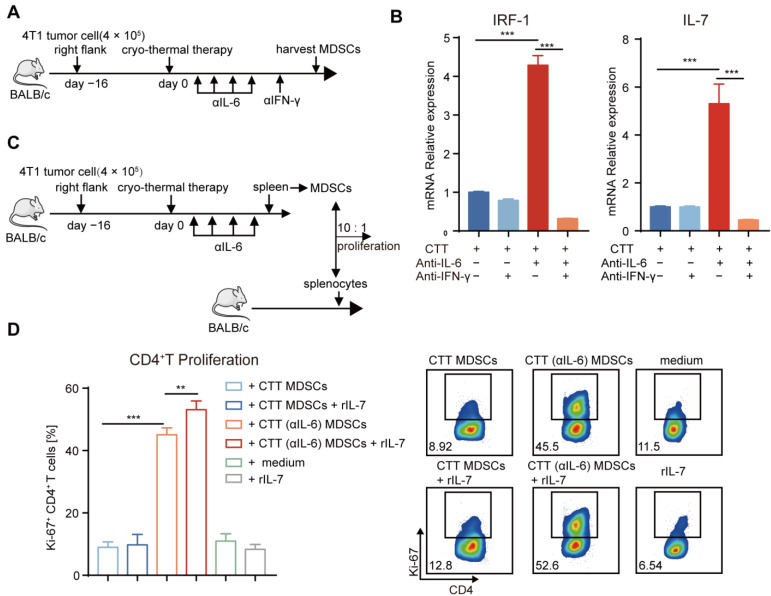
Mature MDSCs after combination therapy promoted the proliferation of CD4^+^ T, which was mediated in part by the IFN-γ/IRF-1/IL-7 axis. (**A**) Experiment schematic: 1 day after combination therapy, mice were treated with anti-IFN-γ (250 μg) and MDSCs were harvested by MACS on Day 21. (**B**) The mRNA level of *IRF1* and *IL-7* was measured by qRT-PCR. (**C**) T cell proliferation assay: splenic MDSCs from different groups were separated by MACS, and then they were co-cultured with splenocytes from age-paired naïve BALB/c mice in ratio of 10: 1 with rIL-7(10 ng/mL) or PBS. (**D**) The expression of Ki-67 in CD4^+^ T cells. *n* = 4 for each group. ** *p* < 0.01, *** *p* < 0.001. Data were analyzed using one-way ANOVA. C, tumor-bearing control; CTT, cryo-thermal therapy.

The aforementioned results indicated that a significant increase in the number of T cells was observed after combination therapy (Figure 4A–D). And the mRNA level of *IL-7* in MDSCs was also increased after combination therapy (Figure 2F), which could promote the proliferation of T cells [39]. Meanwhile, combination therapy could induce differentiation of CD4^+^ T cells to the Th1 phenotype, characterized by the production of IFN-γ [40]. IFN-γ can upregulate the expression of IRF-1 by activating the JAK/STAT1 signaling pathway, which is a transcription factor for IL-7 [41,42]. Then, we hypothesized that the increased proliferative T cells would be mediated by IL-7, which would be regulated by IFN-γ produced by CD4^+^ Th1 cells after combination therapy. To determine whether IFN-γ facilitated the expression of IL-7 in MDSCs, in vivo IFN-γ neutralization was performed after combination therapy and spleen MDSCs were isolated by MACS. Then, the mRNA levels of *IRF-1* and *IL-7* in MDSCs after treatment were measured by qRT-PCR (Figure 7A). The results showed that IFN-γ neutralization abrogated the upregulation of *IRF-1* and *IL-7* in MDSCs induced by combination therapy (Figure 7B), which revealed that IFN-γ could promote the expression of IRF-1 and its downstream cytokine IL-7 in MDSCs after combination therapy.

Then, we identified whether IL-7 promoted T-cell proliferation after combination therapy. Splenic MDSCs from the cryo-thermal and combination therapy groups were incubated with splenocytes from naïve mice in the presence of rIL-7 or PBS for 3 days. The expression level of proliferation-associated nuclear antigen Ki-67 for the evaluation of cell proliferation was assessed by flow cytometry (Figure 7C). The expression level of Ki-67 in CD4^+^ and CD8^+^ T cells was increased after coculture with MDSCs after combination therapy compared with that in cryo-thermal therapy (Figure 7D and Figure S9B). Exogenously administered IL-7 alone did not change the proliferation of CD4^+^ and CD8^+^ T cells (Figure 7D and Figure S9B). However, IL-7 significantly increased the proliferation of CD4^+^ T cells when cocultured with MDSCs from combination therapy but had no effect when cocultured with MDSCs after cryo-thermal therapy (Figure 7D), which suggested that mature MDSCs after combination therapy could induce the proliferation of CD4^+^ T cells partly through IL-7, which could regulate by IFN-γ produced by the CD4^+^ Th1 subset.

Collectively, our studies verified that combination therapy promoted the maturation of MDSCs, leading to Th1-dominant CD4^+^ T immunity. Then, IFN-γ produced mainly by Th1-dominant CD4^+^ T cells activated the IRF1/IL-7 axis in mature MDSCs, potentially promoting CD4^+^ T-cell proliferation and maintaining Th1-dominant antitumor immunity in a positive feedback loop.

## 3. Discussion

In this study, we found that after cryo-thermal therapy, MDSCs produced IL-6 to maintain their immunosuppressive capabilities, impairing the therapeutic effects of treatment. Therefore, cryo-thermal therapy combined with anti-IL-6 treatment was performed in a mouse model of breast cancer, resulting in a significantly improved overall survival rate. Our studies revealed that combination therapy further inhibited the proportion of MDSCs in the spleen and blood and reprogrammed them into a more mature phenotype, ultimately leading to the promotion of the differentiation of Th1-dominant CD4^+^ T cells and enhancing CD8^+^ T-mediated tumor killing. On the other hand, Th1-polarized CD4^+^ T cells further stimulated mature MDSCs to produce IL-7 via the IFN-γ/IRF-1 axis, indirectly contributing to maintaining Th1-dominant antitumor immunity to maximize the therapeutic efficacy of cryo-thermal therapy.

In our study, the primary tumor was entirely ablated after cryo-thermal therapy, so we focused on the change in systemic antitumor immunity. PMN-MDSCs predominate in the spleen and blood of the 4T1 high metastatic breast cancer model and play an important role in modulating systemic antitumor immunity [43]. Moreover, in patients with breast cancer, PMN-MDSCs exceed expansion of other MDSC subsets in the blood [44].

Cryo-thermal therapy alone was insufficient to thoroughly reverse MDSC-mediated immunosuppressive effect, which is crucial contributing factor in the poor outcomes of aggressive tumor models, such as the 4T1 breast cancer model and the Lewis lung cancer model [5,45]. Remarkably, the remaining MDSCs after cryo-thermal therapy facilitated the production of IL-6 through the NF-κB pathway, which could induce the accumulation of MDSCs and sustain MDSC-mediated immunosuppression [46]. To target IL-6, thereby blocking MDSC-mediated immunosuppression, cryo-thermal therapy combined with anti-IL-6 treatment was performed in mice bearing 4T1 breast cancer and Lewis lung cancer (Figure 2A,B and Figure S2)*,* contributing to a significantly improved long-term survival rate compared to cryo-thermal therapy alone.

Cryo-thermal therapy alone could reduce the proportion of MDSCs and promote the expression of MHC II and CD86 on MDSCs, but the NF-κB pathway in MDSCs was also enriched, resulting in a high level of IL-6 after treatment, which indicated that cryo-thermal therapy could not maximally reverse immunosuppression induced by MDSCs in 4T1 breast cancer model. A highly aggressive 4T1 breast cancer model could spontaneously metastasize and induce an MDSC-dominated immunosuppressive environment [47,48,49]. The remaining 4T1 cells could promote residual MDSCs after cryo-thermal therapy to produce IL-6 through G-CSF [38,50], in turn, IL-6 participated in the accumulation and immunosuppressive function of MDSCs. Cryo-thermal therapy combined with anti-IL-6 treatment reprogrammed MDSCs toward a more mature phenotype; in particular, the mRNA level of IL-7 in mature MDSCs after combination therapy was significantly increased. IL-7 could promote T-cell proliferation [39], in line with our results that exogenous IL-7 further promoted the proliferation of CD4^+^ T cells in the presence of mature MDSCs after combination therapy. However, in the presence of incompletely mature MDSCs after cryo-thermal therapy, the additional IL-7 did not have a significant pro-proliferative effect on CD4^+^ T cells, suggesting that the antitumor function of IL-7 was only evident following the reversal of immunosuppression induced by MDSCs, which could explain why the addition of IL-7 alone did not significantly improve survival of tumor-bearing mice with immunosuppressive environment [39]. Therefore, the pro-proliferative effect of IL-7 on CD4^+^ T cells would be indirect, and other soluble factors secreted by mature MDSCs or intercellular contact interactions between mature MDSCs and CD4^+^ T cells would trigger the pro-proliferative effect of IL-7 [51,52]. Consequently, cryo-thermal therapy combined with IL-7 treatment could potentially improve the outcomes of tumor models with less MDSC accumulation.

CD4^+^ Th1 cells play a critical role in endogenous systematic antitumor immunity by promoting the proliferation of CD8^+^ T cells, enhancing the cytotoxic functions of CD8^+^ T cells and NK cells, driving M1 macrophage polarization and promoting the maturation of MDSCs [53,54]. However, IL-6 was responsible for impairing CD4^+^ T-mediated antitumor immunity, which inhibited CD4^+^ Th1 development through the c-MAF/IL-4/IL-21 axis, resulting in a reduced CD4^+^ T-cell helper effect on cytotoxic CD8^+^ T cells [55]. Furthermore, IL-6 led to the differentiation of CD4^+^ T cells toward the Th17 phenotype in conjunction with TGF-β [56], resulting in an immunosuppressive environment [57]. IL-6 can induce the mRNA expression of Bcl6, thereby promoting the differentiation of Tfh cells in vivo [58]. Although cryo-thermal therapy promoted the differentiation of CD4^+^ T cells into Th1 subsets, the Th1 subset was not dominant in the differentiation of CD4^+^ T cells after treatment because the levels of Th17 and Tfh cells were also increased. In contrast, combination therapy could further promote the differentiation of CD4^+^ T cells toward a Th1-dominant pattern and inhibit differentiation toward Th17 and Tfh subsets. Therefore, we suggest that Th1-dominant CD4^+^ T-cell immunity induced after combination therapy is attributed to blocking IL-6-mediated immunosuppression induced after cryo-thermal therapy.

IL-6 could induce immunosuppressive characteristics of MDSCs by inducing the upregulated expression of PD-L1 and Arg-1 on MDSCs, promoting ROS and NO production, and downregulating the antigen presentation function of MDSCs [17]. Thus, IL-6 blockade is considered a potential immunotherapeutic strategy targeting MDSCs. However, anti-IL-6 treatment alone cannot change the complex immune system, leading to poor clinical outcomes in several highly aggressive tumors [18]. Cryo-thermal therapy can elicit systematic antitumor immunity [53]. However, in the 4T1 highly aggressive tumor model, it was insufficient to reverse MDSC-mediated immunosuppression because of the IL-6 signaling pathway in MDSCs. Therefore, cryo-thermal therapy in conjunction with anti-IL-6 treatment in highly aggressive and MDSC-dominated immunosuppressive tumor model could attain a superior therapeutic outcome. We suggest that as cryo-thermal therapy can remodel organism immunity, it can serve as a platform technology to combine with other antitumor drugs, leading to further improvement of antitumor immunity for achieving long-term survival of tumor-bearing mice with MDSC-dominated immunosuppressive environment.

In conclusion, combining cryo-thermal therapy with anti-IL-6 treatment induced CD4^+^ Th1-dominant immunity to achieve long-term survival of breast cancer-bearing mice with an MDSC-dominated immunosuppressive environment. Our studies not only shed light on the efficiency of combination therapy but also present a promising and feasible combined therapeutic strategy targeting highly MDSC-dominated immunosuppressive tumors in clinical trials.

## 4. Materials and Methods

### 4.1. Cell Culture and Murine Cancer Models of Breast Cancer

The murine 4T1 line (triple negative breast cancer) was obtained from Shanghai First People’s Hospital, Shanghai, China. 4T1 cells were cultured in DMEM medium with 10% FBS (Gemini Bio-Products, West Sacramento, CA, USA) and 100 units/mL penicillin/streptomycin (Hyclone, Logan, UT, USA). Wild-type female BALB/c mice were obtained from the Shanghai Slaccas Experimental Animal Co., Ltd. (Shanghai, China). To prepare the tumor-bearing mice, 4 × 10^5^ 4T1 cells were injected subcutaneously into the right femoral region of BALB/c mouse. All animal experiments were accepted by Shanghai Jiao Tong University’s Animal Welfare Committee, and all experimental methods followed the Shanghai Jiao Tong University Animal Care Guidelines (approved by Shanghai Jiao Tong University Scientific Ethics Committee, Proto code 2020017, 26 March 2020).

### 4.2. The Cryo-Thermal Therapy and Anti-IL-6 Treatment Procedures

Tumor size was assessed regularly with vernier caliper using the formula *V* (cm^3^) = width (cm) × length (cm) × height (cm) × π/6. When tumor volume reached 0.2 cm^3^ (nearly 16 days), the cryo-thermal therapy system which is maintained by Dr. Aili Zhang and Engineer Jincheng Zou in our laboratory was performed. The treatment procedures include (1) liquid nitrogen freezing; (2) natural rewarming; (3) radiofrequency ablation. The detail steps were described in our previous studies [20]. Before cryo-thermal treatment, mice were anesthetized with 1.6% pentobarbital sodium (0.5 mL/100 g, Sigma-Aldrich, St. Louis, MO, USA). On Day 7 after cryo-thermal therapy, treatment with IgG (HRPN, Bio X Cell, West Lebanon, NH, USA), anti-IL-6 (MP5-20F3, Bio X Cell, West Lebanon, NH, USA), or a combination therapy consisting of cryo-thermal therapy and anti-IL-6 treatment was initiated. For anti-IL-6 treatment, mice bearing 4T1 murine breast cancer were injected intraperitoneally with 20 μg of anti-IL-6 mAb every four days for a total of four injections on Day 7 after cryo-thermal therapy.

### 4.3. Flow Cytometry Analysis

Mice were sacrificed to obtain splenocytes and blood cells, and erythrocytes were lysed using erythrocyte lysis solution (0.15 M of NH_4_Cl, 1.0 M of KHCO_3_, and 0.1 mM of Na_2_EDTA), the cells were filtered using a 70 μm cell strainer to gain single cell suspension. Cells were resuspended in PBS and incubated with different fluorescently labeled antibodies purchased from Biolegend, BD Biosciences and eBioscience, which are listed in Supplementary Table S1. For dead cells exclusion, Zombie Aqua (Biolegend, San Diego, CA, USA) was used. To measure intracellular cytokines, the cells were resuspended in complete RPMI 1640 medium (Cytiva, Marlborough, MA, USA) and stimulated with cell activation cocktail (Biolegend, San Diego, CA, USA) in a 5% CO_2_ incubator at 37 °C for 4 h. After cell-surface staining, cells were fixed and permeabilized by using fixation buffer (Biolegend, San Diego, CA, USA) and permeabilization wash buffer (Biolegend, San Diego, CA, USA), then stained by intercellular cytokine antibodies. For transcription factor detection, true-nuclear transcription factor buffer set (Biolegend, San Diego, CA, USA) was used. Samples were analyzed with BD FACS Aria II cytometer (BD Biosciences, Franklin Lakes, NJ, USA) and the results were analyzed using FlowJo software (version 10.8.2). Gating strategy by flow cytometry was shown in Figure S1A, B, and representative plots of the negative control and experimental samples of flow cytometry were shown in Figure S1C.

### 4.4. Serum Collection and ELISA

Serum of naïve BALB/c mice and tumor-bearing mice treated with cryo-thermal therapy was collected, and the cytokines TGF-β, IL-10 and IL-6 were detected by ELISA Kit (Boster Biological Technology, Wuhan, China) according to the manufacturer’s instructions.

### 4.5. In Vitro Co-Culture Assay

MDSCs from the spleen were first incubated with Gr1-PE and then isolated using PE Positive Selection Kit (StemCell Technologies, Vancouver, BC, Canada), and cells with purity of >80% were used for experiments. For functional studies, MDSCs were co-cultured in a 1:1 ratio with splenocytes from tumor-bearing control mice (without MDSCs, 16 days after tumor inoculation) for 24 h, the level of IFN-γ, IL-4, IL-17, IL-21, granzyme B and perforin were assessed by flow cytometry. For T cell proliferation assays, splenocytes from naïve mice were obtained and stimulated with anti-CD3 (1 ng/mL, Biolegend, San Diego, CA, USA), after co-culture with MDSCs isolated from different groups in the E/T ratio of 10:1 for 3 days as reported by Jimenez et al. [59], T-cell proliferation was measured by the level of Ki-67 (gated on CD3) using BD LSRFortessa cytometer (BD Biosciences, Franklin Lakes, NJ, USA).

### 4.6. Tumor Cell Killing Assay

4T1 cells were labeled with calcein-AM in advance. Splenic CD4^+^ T and CD8^+^ T cells isolated using CD4^+^ T Cell Positive Selection Kit (StemCell Technologies, Vancouver, BC, Canada) and CD8^+^ T Cell Isolation Kit (StemCell Technologies, Vancouver, BC, Canada) were plated in a 96-well plate, then co-cultured with 4T1 cells at ratio of 10:1 as reported by Mishra et al. and Ning et al. [60,61]. Cells were cultured in complete RPMI 1640 medium at 37 °C in a 5% CO_2_ incubator for 6 hours. The fluorescence level of calcein in the supernatant was used to detect the killing ratio of 4T1 cells in the formula: killing ratio% = [(experimental group − free release group)/(positive control group − free release group)] × 100%.

### 4.7. RNA Isolation and qRT-PCR

Total RNA from isolated MDSCs was obtained using RNAiso reagent (TaKaRa, Otsu, Shiga, Japan) according to the manufacturer’s instructions. The concentration of extracted RNA was detected by nanodrop and reversed transcribed to cDNA using the PrimeScript RT Reagent Kit (TaKaRa, Otsu, Shiga, Japan). Quantitative reverse transcription PCR (qRT-PCR) was quantified using SYBR Green PCR Master Mix (Yeasen, Shanghai, China) with ABI 7900HT sequence detection system. All the primers were listed in Supplementary Table S2. Relative mRNA level of target genes based on their Ct values normalized to the Ct value of GAPDH (reference gene) using the 2^−ΔΔCt^ method. The experiment was repeated three times.

### 4.8. Analysis of RNA-Seq

RNA-seq data were obtained from our previous study [28]. Gene Set Enrichment Analysis (GSEA) was performed using GSEA software [62]. *p* value < 0.05 and fold change > 2 or fold change < 0.5 was set as the threshold for significantly differential expression. Hierarchical cluster analysis of DEGs was performed to explore gene expression patterns. KEGG [63] pathway enrichment analyses of DEGs were respectively performed using R based on the hypergeometric distribution.

### 4.9. Statistical Analysis

Survival rate of mice with different treatments were compared using the Kaplan–Meier method and the log-rank test. The other outcomes were compared using one-way ANOVA (comparing three or more experimental groups) and two-sided Student’s *t*-test (comparing two experimental groups). These analyses were performed by GraphPad Prism 7.0 software (GraphPad, La Jolla, CA, USA). Data were presented as mean ± SD. *p* values < 0.5 were regarded as significant (* *p* < 0.05, ** *p* < 0.01, *** *p* < 0.001).

## Data Availability

Data are contained within the article or Supplementary Materials or are available on request from the corresponding author.

## Supplementary Materials

The following are available online at https://www.mdpi.com/article/10.3390/ijms24087018/s1.
