# Supplementary material for: Combining Cryo-Thermal Therapy with Anti-IL-6 Treatment Promoted the Maturation of MDSCs to Induce Long-Term Survival in a Mouse Model of Breast Cancer
Source: Int J Mol Sci. 2023 Apr 10;24(8):7018. doi: 10.3390/ijms24087018 (PMC10138396; doi:10.3390/ijms24087018)
Supplement: Supplementary file 1 [file ijms-24-07018-s001.zip › ijms-2300260-supplementary.pdf]

Supplementary Material

Supplementary Figures

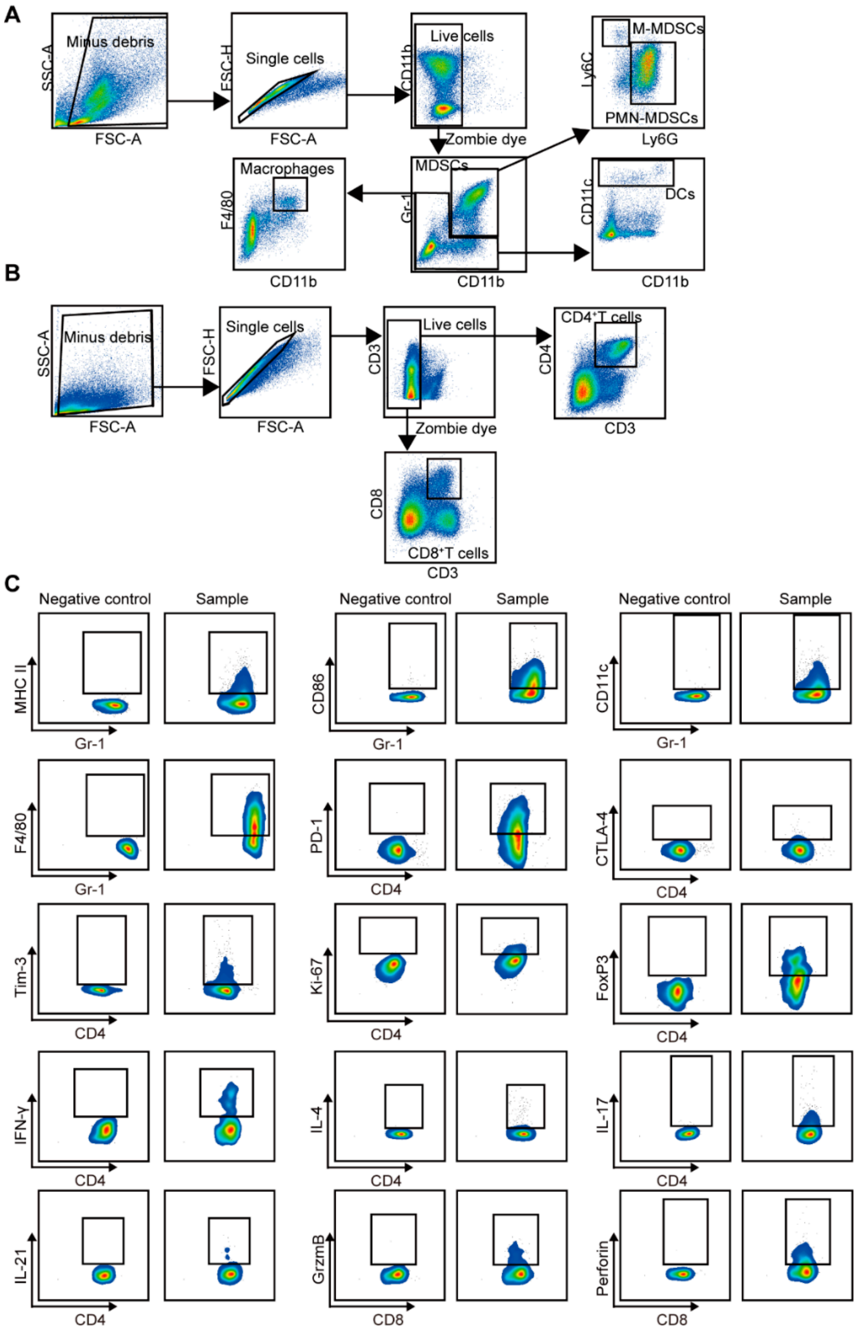

Figure S1. Gating strategy of flow cytometry. (**A**,**B**) Single-cell suspensions of splenocytes were stained with fluorescence conjugated antibodies, gating strategy of myeloid-derived cells (**A**) and T cells (**B**). (C) Representative plots of the negative control and experimental samples of flow cytometry.

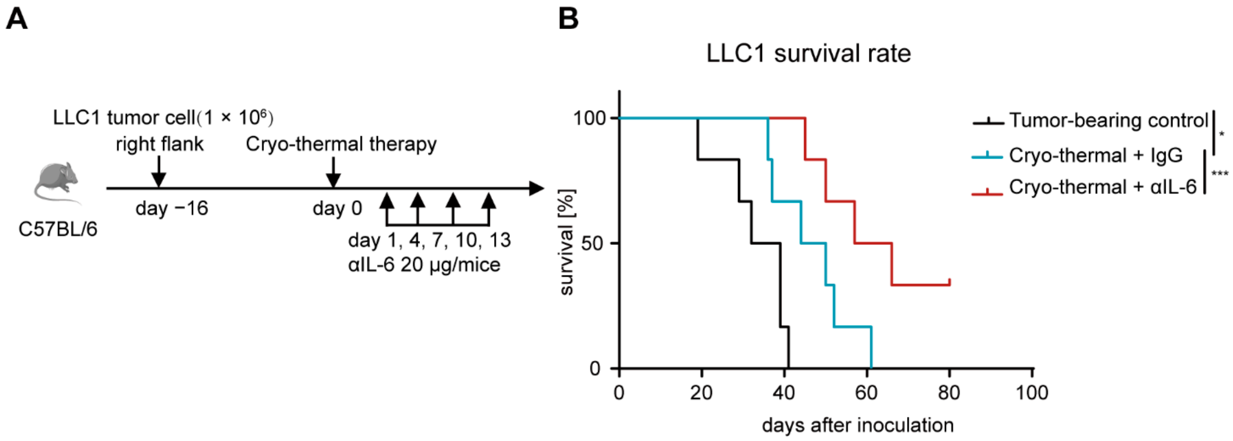

Figure S2. Combination therapy prolonged the long-term survival of LLC1 lung cancer-bearing mice. (**A**) Experiment schematic: 1 × 10^6 LLC1 tumor cells were injected subcutaneously in the right flanks of wild-type C57BL/6 mice, after 12 days, tumor-bearing mice were treated with cryo-thermal therapy. 1 day after cryo-thermal therapy, mice were treated with 20 μg (intraperitoneal injection one time/3 days) anti-IL-6 mAb, or the same dose of IgG1. (**B**) A Kaplan-Meier survival curve showed the Lewis lung cancer model either treated with cryo-thermal therapy or combination therapy, n=6 per cohort, log-rank tests were used to compare survival curve. * $p < 0.05$, *** $p < 0.001$.

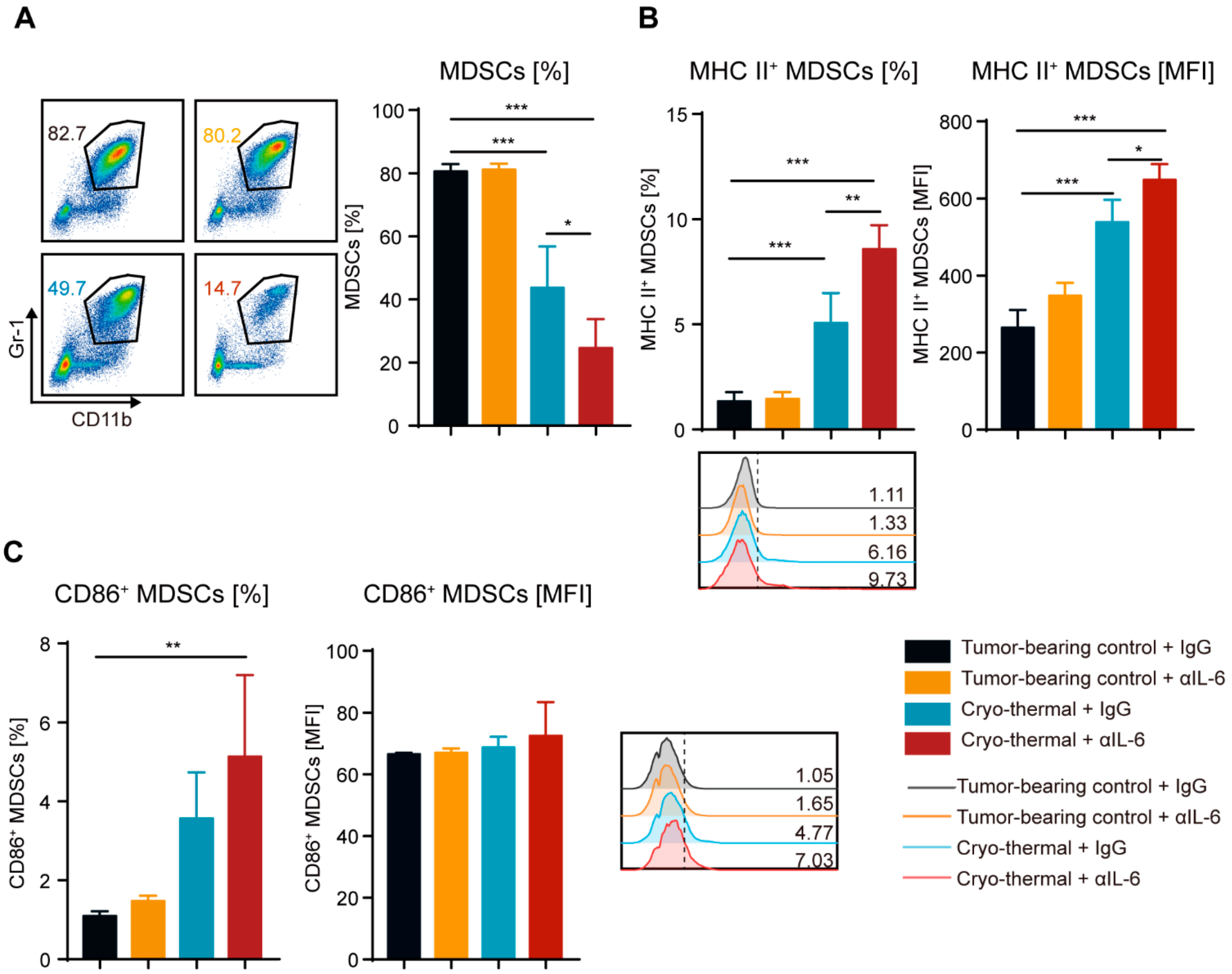

Figure S3. Combination therapy decreased the proportion of MDSCs and promoted the maturation of MDSCs in the blood. (**A**) The proportion of blood MDSCs. (**B,C**) The expression of MHC II⁺ (**B**) and CD86⁺ (**C**) MDSCs in the bloof. In the representative graph of flow cytometry, black represent tumor-beaing control + IgG, orange represent tumor-beaing control + αIL-6, blue represent cryo-thermal + IgG, red represent cryo-thermal + αIL-6. n=4 for each group. ** P<0.01, *** P<0.001. Data were analyzed using one-way ANOVA.

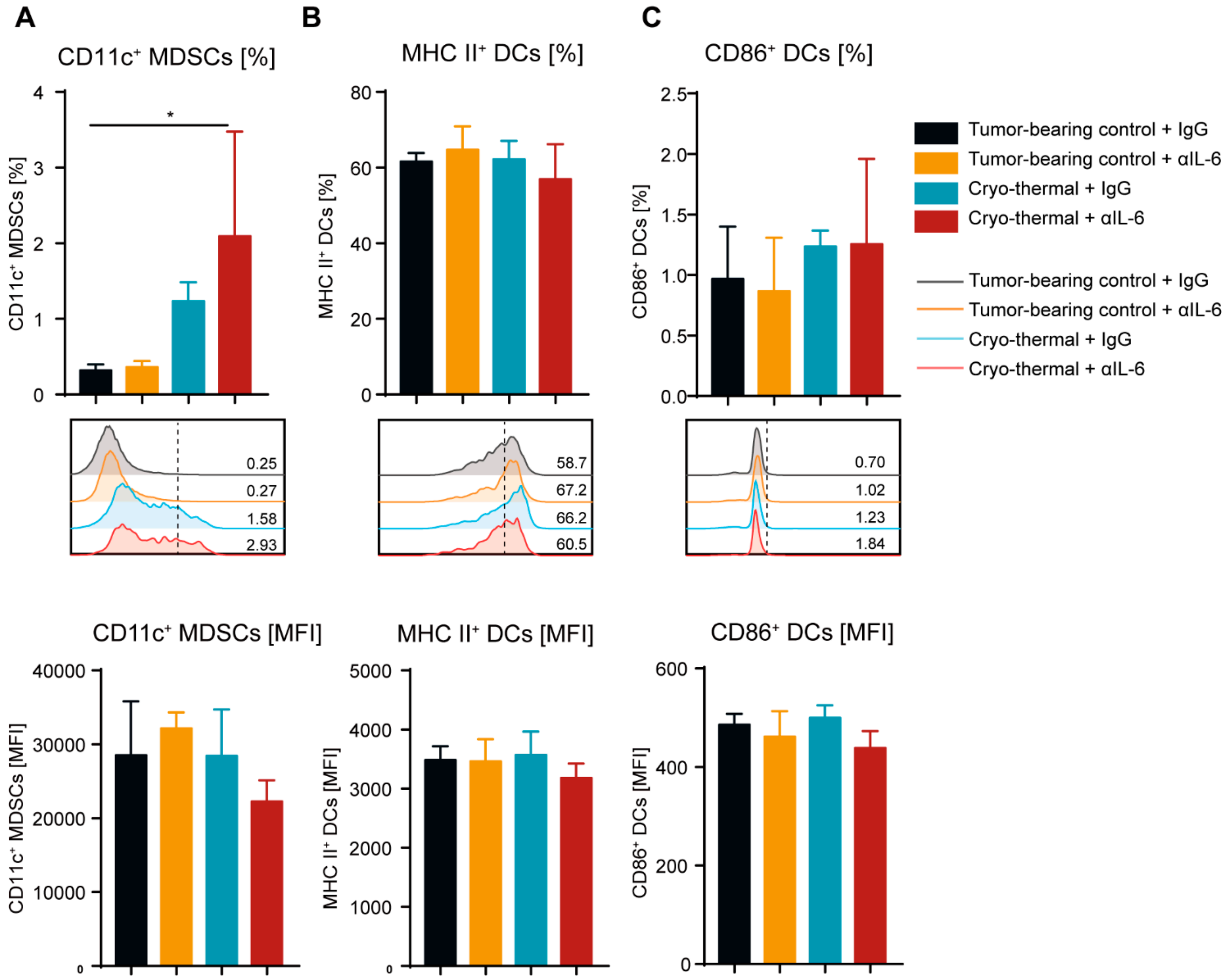

Figure S4. Combination therapy induced the differentiation of MDSCs to DCs. (**A**) The expression of CD11c on splenic MDSCs. (**B,C**) The expression of MHC II⁺ (**B**) and CD86⁺ (**C**) DCs in the spleen was detected by flow cytometry. n=4 for each group. * $p < 0.05$. Data were analyzed using one-way ANOVA.

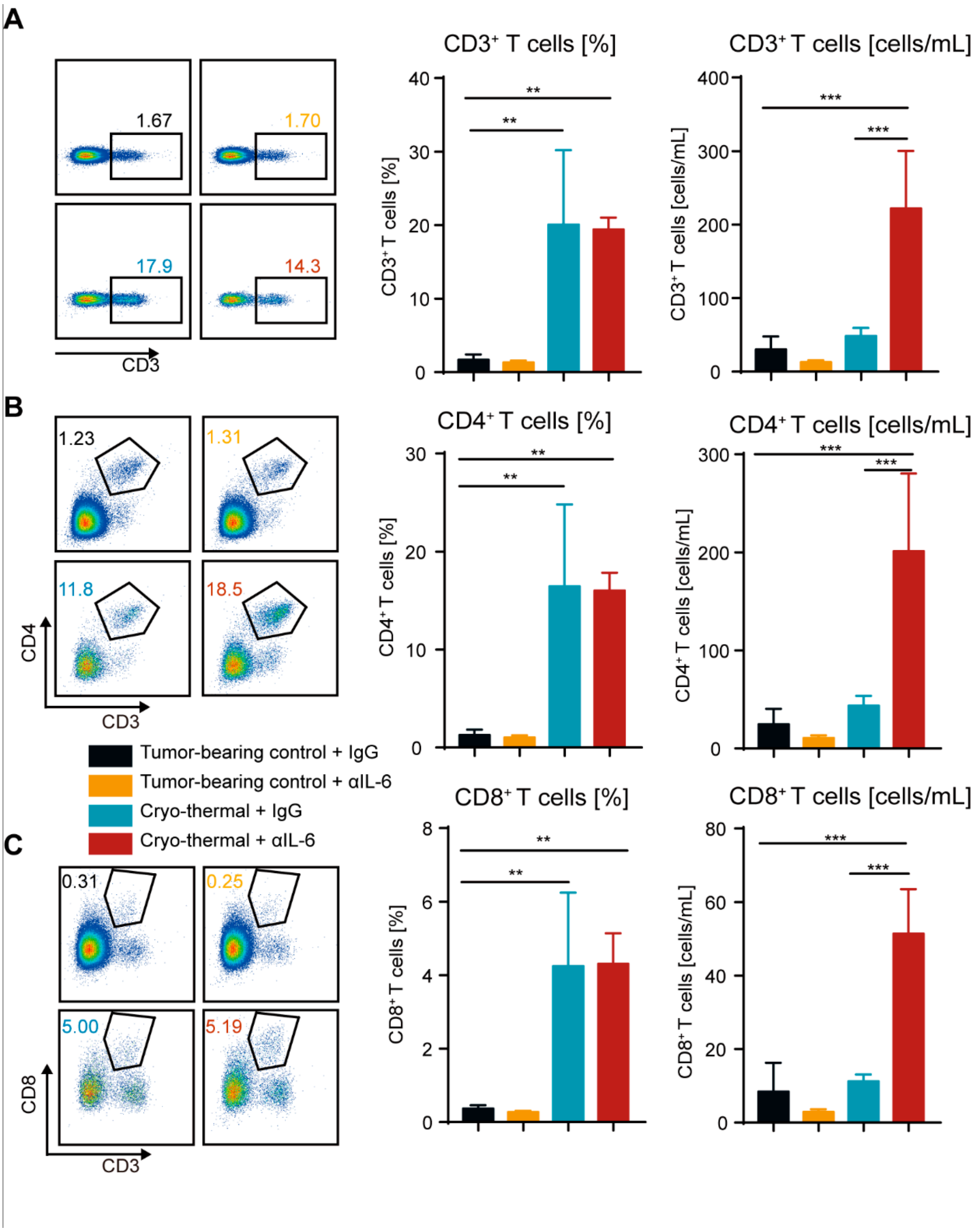

Figure S5. The numbers of blood CD3⁺, CD4⁺ and CD8⁺ T cells after combination therapy were increased. (**A**–**C**) Flow cytometry analyzed the percentage and absolute number of blood CD3⁺ T cells (**A**), CD4⁺ T cells (**B**), and CD8⁺ T cells (**C**). In the representative graph of flow cytometry, black represent tumor-beaing control + IgG, orange represent tumor-beaing control + αIL-6, blue represent cryo-thermal + IgG, red represent cryo-thermal + αIL-6. n=4 for each group. ** $p < 0.01$, *** $p < 0.001$. Data were analyzed using one-way ANOVA.

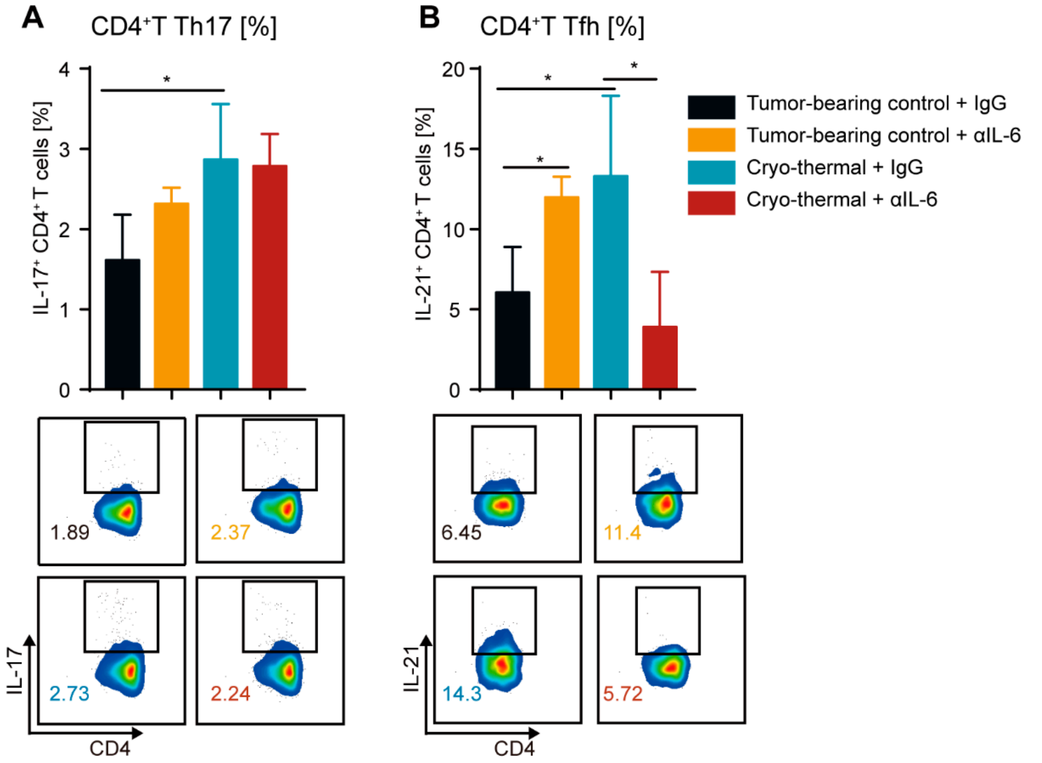

Figure S6. The percentage of Th17 and Tfh cells was reduced after combination therapy. (**A**,**B**) Flow cytometry analyzed the percentage of splenic Th17 (**A**) and Tfh (**B**). In the representative graph of flow cytometry, black represent tumor-beaing control + IgG, orange represent tumor-beaing control + αIL-6, blue represent cryo-thermal + IgG, red represent cryo-thermal + αIL-6. n=4 for each group. * $p < 0.05$. Data were analyzed using one-way ANOVA.

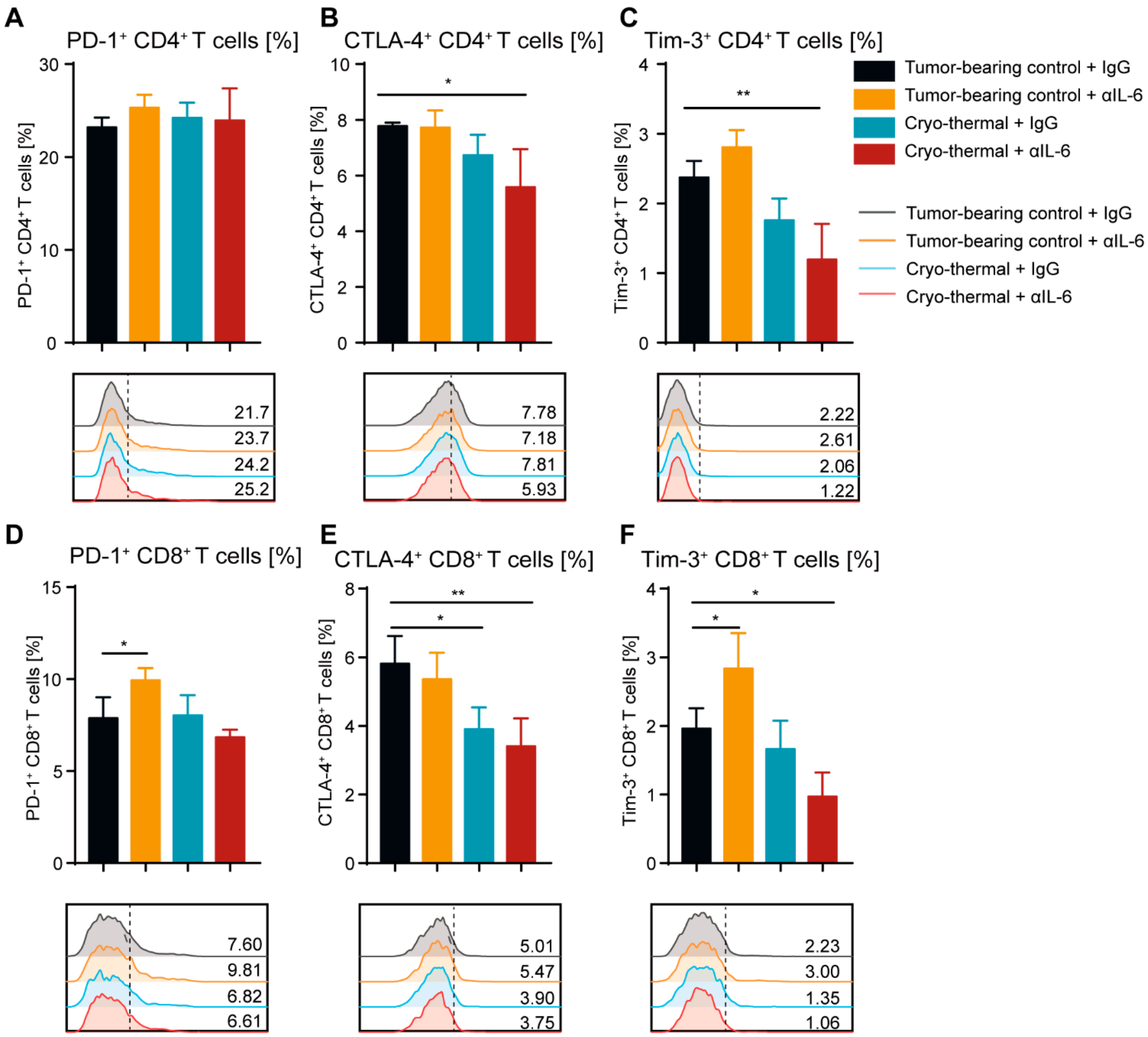

Figure S7. Combination therapy inhibited CD4⁺ T cells exhaustion. (**A–C**) The frequency of PD-1⁺ CD4⁺ T cells (**A**), CTLA4⁺ CD4⁺ T cells (**B**), and Tim-3⁺ CD4⁺ T cells (**C**) in the spleen. (**D–F**) The frequency of PD-1⁺ CD8⁺ T cells (**D**), CTLA4⁺ CD8⁺ T cells (**E**), and Tim-3⁺ CD8⁺ T cells (**F**) in the spleen was measured by flow cytometry. n=4 for each group. * $p < 0.05$, ** $p < 0.01$. Data were analyzed using one-way ANOVA.

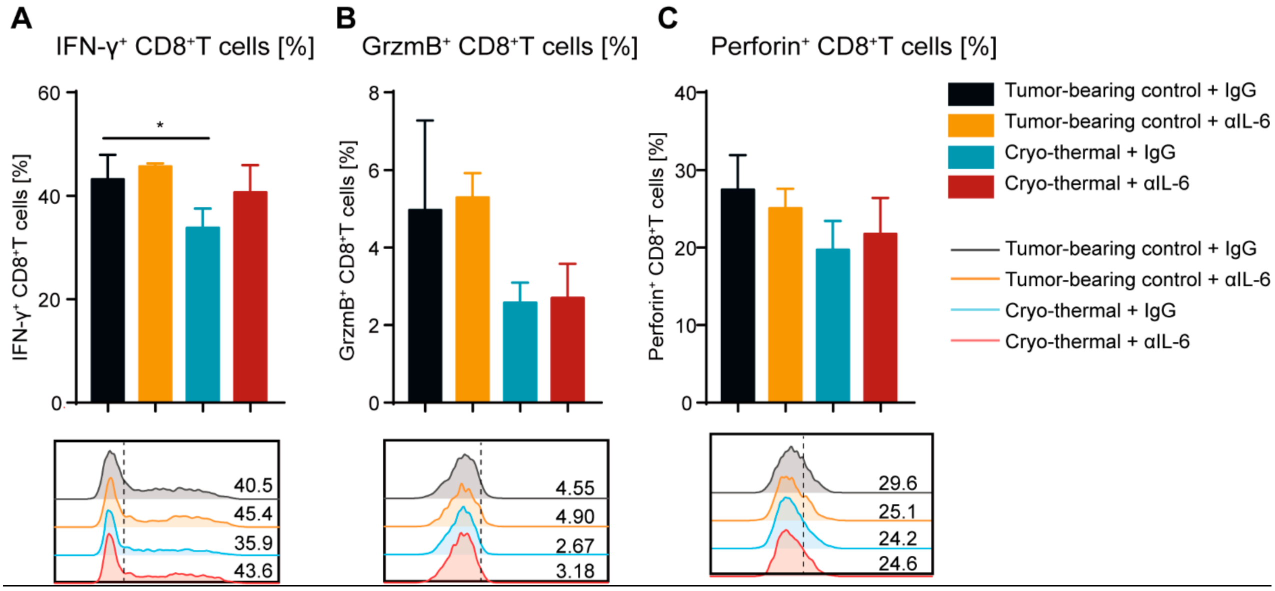

Figure S8. Combination therapy did not change the frequency of CD8⁺ CTLs. (**A–C**) The frequency of IFN-γ⁺ CD8⁺ T cells (**A**), grzmB⁺ CD8⁺ T cells (**B**), and perforin⁺ CD8⁺ T cells (**C**) in the spleen was measured by flow cytometry. n=4 for each group. * $p < 0.05$, ** $p < 0.01$. Data were analyzed using one-way ANOVA.

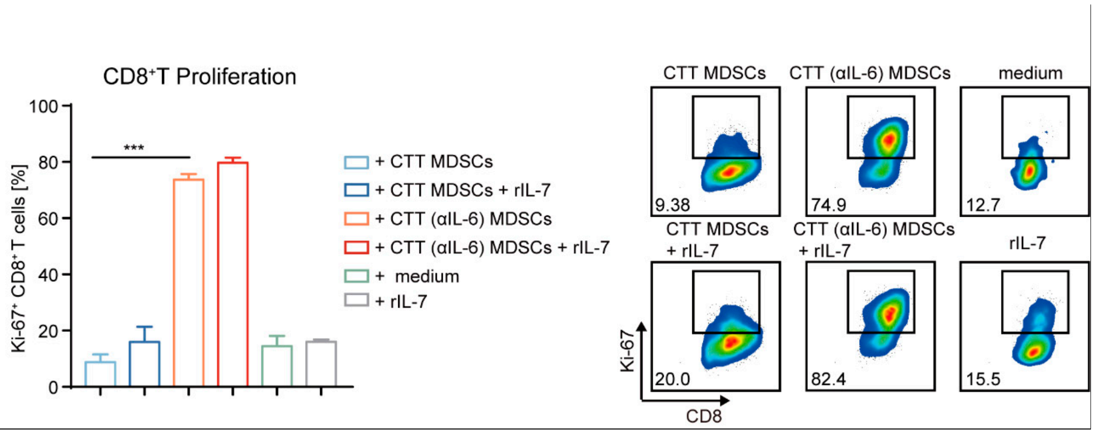

Figure S9. Combination therapy promoted the proliferation of CD8+ T cells. The expression of Ki-67 in CD8+ T cells. n=4 for each group. *** $p < 0.001$. Data were analyzed using one-way ANOVA.

Supplementary Table

Supplementary Table S1. Primer sequences of various genes in this study.

Name	Primer Sequence (5′-3′)
IL-7-F	TTCCTCCACTGATCCTTGTTCT
IL-7-R	AGCAGCTTCCTTTGTATCATCAC
IL-12-F	TGGTTTGCCATCGTTTTGCTG
IL-12-R	ACAGGTGAGGTTCACTGTTTCT
IL-15-F	AGAGGCCAACTGGATAGATGT
IL-15-R	AGAGCACGTTTCTTACTGTTTCA
IL-6-F	GACAAAGCCAGAGTCCTTCAGAGAGATACAG
IL-6-R	TTGGATGGTCTTGGTCCTTAGCCAC
TNFα-F	TTCTGTCTACTGAACTTCGGGGTGATCGGTCC
TNFα-R	GTATGAGATAGCAAATCGGCTGACGGTGTGGG
IL-10-F	GCTCTTACTGACTGGCATGAG
IL-10-R	CGCAGCTCTAGGAGCATGTG
Arg-1-F	TTGGGTGGATGCTCACACTG
Arg-1-R	GTACACGATGTCTTTGGCAGA
IL-1β-F	ACAGCAGCACATCAACAAGAG
IL-1β-R	ATGGGAACGTCACACACCAG
PD-L1-F	GCTTCTCAATGTGACCAGCA
PD-L1-R	GAGGAGGACCGTGGACACTA
iNOS-F	ACATCGACCCGTCCACAGTAT
iNOS-R	CAGAGGGGTAGGCTTGTCTC
TGF-β-F	CTCCCGTGGCTTCTAGTGC
TGF-β-R	GCCTTAGTTTGGACAGGATCTG

Supplementary Table S2. Antibodies used in the flow cytometry analysis.

Antibodies	Fluorescence Labeling	Clone	Company
CD11b	FITC	M1/70	Biolegend
CD11b	Pacific blue	M1/70	Biolegend
Gr-1	PE	RB6-8C5	Biolegend
Gr-1	APC-Cy7	RB6-8C5	Biolegend
Ly6C	FITC	HK1.4	Biolegend
Ly6G	PE-Cy7	1A8	Biolegend
F4/80	APC	BM8	Biolegend
CD86	APC-Cy7	GL-1	Biolegend
MHC-II	Percp-Cy5.5	M5/114.15.2	Biolegend
CD3	Percp-Cy5.5	145-2C11	Biolegend
CD4	APC-Cy7	RM4-5	Biolegend
CD25	PE	3C7	Biolegend
CD19	BV510	6D5	Biolegend
PD-1	PE-Cy7	29F.1A12	Biolegend
CTLA-4	PE	UC10-4B9	eBioscience
Tim-3	BV421	RMT2-23	Biolegend
IFN-γ	PE/Dazzle 594	XMG1.2	BD Biosciences
IL-4	BV421	11B11	Biolegend
IL-6	PE	MP5-20F3	Biolegend
IL-17	PE	TC11-18H10.1	Biolegend
IL-21	APC	FFA21	eBioscience
Granzyme B	APC	GB11	Biolegend
Perforin	PE	S16009A	Biolegend
FoxP3	PE-CF594	MF23	BD Biosciences
Ki-67	PE-cy7	16A8	Biolegend
